# Locking bandwidth of two laterally-coupled semiconductor lasers subject to optical injection

**DOI:** 10.1038/s41598-017-18379-7

**Published:** 2018-01-08

**Authors:** Nianqiang Li, H. Susanto, B. R. Cemlyn, I. D. Henning, M. J. Adams

**Affiliations:** 1 School of Computer Science and Electronic Engineering, University of Essex, Wivenhoe Park, Colchester, CO4 3SQ United Kingdom; 2 Department of Mathematical Sciences, University of Essex, Wivenhoe Park, Colchester, CO4 3SQ United Kingdom

## Abstract

We report here for the first time (to our knowledge), a new and universal mechanism by which a two-element laser array is locked to external optical injection and admits stably injection-locked states within a nontrivial trapezoidal region. The rate equations for the system are studied both analytically and numerically. We derive a simple mathematical expression for the locking conditions, which reveals that two parallel saddle-node bifurcation branches, not reported for conventional single lasers subject to optical injection, delimit the injection locking range and its width. Important parameters are the linewidth enhancement factor, the laser separation, and the frequency offset between the two laterally-coupled lasers; the influence of these parameters on locking conditions is explored comprehensively. Our analytic approximations are validated numerically by using a path continuation technique as well as direct numerical integration of the rate equations. More importantly, our results are not restricted by waveguiding structures and uncover a generic locking behavior in the lateral arrays in the presence of injection.

## Introduction

Coupled nonlinear oscillators/systems have received considerable attention due to their rich dynamics including stable continuous wave (cw) operation, oscillatory states and chaos, as well as collective dynamical behavior, e.g. synchronization of periodic and even chaotic oscillations; see, e.g.^1–3^, and references therein. One relatively simple example of these systems is the 1-dimensional (1-D) lateral laser array with nearest-neighbor interactions (or evanescently-coupled laser array)^4^. Researchers are interested in this basic array because practically it can be readily fabricated on a single chip, and also it can be accurately modelled by a set of ordinary differential equations, usually called the coupled laser model. This basic set of simple rate equations makes extending the investigation to larger 1-D or even 2-dimensional arrays more tractable and, when coupled with a detailed bifurcation analysis, regions of stability and dynamics and their nature can be revealed. In particular, the study of lateral laser arrays has been motivated, among other things, from the perspective of engineering applications, for instance, by developing designs and implementing appropriate technology to obtain stable high-power operation in a narrow beam^5,6^, as well as enabling significant modulation bandwidth enhancement^7,8^. Aside from its technological applications, from the physics viewpoint the investigation of such a configuration has fundamental interest and can clarify the complex dynamical behaviour as mentioned above (see^9^ and references therein). Furthermore, these devices turn out to be sources for uncovering novel physical phenomena such as gain tuning and parity-time symmetry breaking^10^, turbulent chimeras^11^, as well as a periodicity of behavior with laser separation^12^. Among many others, two laterally coupled lasers forming the simplest laser array (hereafter, a two-element laser array) have been widely studied both theoretically^7,12–18^ and experimentally^8,10,19–21^. In particular, the excellent agreement between experimental measurements and coupled-mode theory in laser arrays in^10^ is expected to stimulate more research in this fruitful field.

Stable locking behavior is a generic property in many coupled systems^22–26^, and lateral laser arrays are no exception^12,17–19^. Previous bifurcation analyses have shown that stable locking regions can be found in two- and three-element laser arrays and depend strongly on the degree of amplitude-phase coupling of the lasing field that is characterized by the linewidth enhancement factor^12,18,27^. Regions of the parameter space in which either in-phase or out-of-phase cw states exist can be easily determined; however, in laser arrays, more complicated dynamical states are dominant^12,18,27–29^. Interestingly, some feedback configurations, such as feedback on the bias current or an external mirror, can be used to stabilize laser arrays^30–33^. A commonly adopted approach is locking the elements by introducing an external input (i.e. a master laser operating in a cw regime)^34,35^. An important advantage of this approach is that it is a very effective means to improve the performance of semiconductor lasers through resonance frequency enhancement^36^, frequency chirp reduction^37^ and laser spectral narrowing^38^. The simplest model for this type of system is a single semiconductor laser (slave) subject to optical injection from a master laser^39^. Here the stable locking conditions are well understood: the stable region is bounded by a saddle-node (SN) and a Hopf bifurcation line, and outside of this region the system exhibits a wealth of dynamical behaviour which can include pulsations, chaos, periodic oscillations and multi-stability^34,40^. A similar injection-locking technique has been applied to broad-area laser arrays^41^ and other laser arrays consisting of conventional edge-emitting lasers^42–45^ or vertical-cavity surface-emitting lasers^46,47^. For example, the control of the far-field beam pattern and the spectrum of 10-element laser arrays by injection locking to a single-mode master laser has been experimentally demonstrated, and similar results can be achieved when the entire array is illuminated by the master laser beam or when only one of the elements is illuminated^42^. Moreover, the phase-locked solution in two-element arrays can be stabilized at a low external injection-locking power with a suitably chosen frequency detuning between the laser array and the master laser^48^. Despite the large amount of theoretical and experimental work^35,41–48^ the actual mechanisms of how optical injection induces stable injection locking and the underlying bifurcation boundaries of the locking range in laser arrays have never been analyzed.

The present work has been prompted by our recent study of waveguide properties on the dynamics of a two-element array whose lasers are coupled by means of their overlapping evanescent fields^12^. The result of that report was to reveal a previously overlooked periodicity of behavior with laser separation. It was shown that this periodicity has increasing influence on the bifurcations of the system as the structures develop from those with purely real guidance to a combination of index antiguiding and gain-guiding. Here we extend the model discussed in^12^ by introducing optical injection into one element of the array and give a detailed and comprehensive analysis of locking conditions in this system. Our purpose is to determine the locking range and to develop an analytic approximation for the conditions that define the domain of stable locking. In order to validate the approximation, we perform a bifurcation analysis using the standard continuation package AUTO^49^, complemented by numerical solution of the rate equations. Additionally, we consider the influence of the four waveguide systems introduced in^12^ and some key parameters, including linewidth enhancement factor, laser separation and frequency offset (frequency difference between the two waveguide lasers), on the locking range and width.

## Results

### Formulation

**Figure 1:**
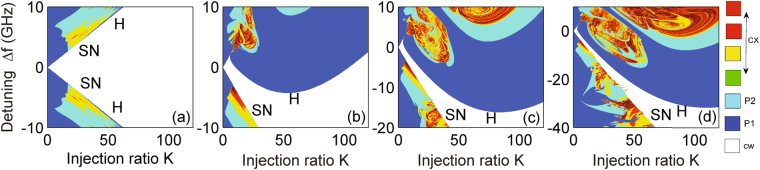
Stability map of a single laser with injection in the (*K*, Δ*f*) plane for four different representative values of the linewidth-enhancement factor *α*_*H*_: (**a**) *α*_*H*_ = 0, (**b**) 2, (**c**) 3, and (**d**) 4. H and SN stand for the Hopf and saddle-node bifurcations, respectively. The white region denotes stable operation (cw), dark blue represents P1, light blue stands for P2, while other colors (from green to yellow, red, and dark red) refer to complex dynamics (cx).

Our basic model is a two-element laser array which consists of two laterally-coupled semiconductor lasers, i.e., two identical laser waveguides, A and B, each of width 2*a*, with an edge-to-edge separation of 2*d*, as illustrated schematically in Fig. 1 in^12^. As detailed in that paper, we have modelled the laser system by a set of ordinary differential equations, which provide a basis for the current study. Here we consider a basic master-slave setup in which only laser A (guide A) of the two-element array is subject to external optical injection. Following^12^ with a straightforward modification to account for the optical injection we restrict ourselves to the case where a solitary laser supports a single transverse mode and extend the basic coupled-mode equations to include an externally injected field *k*_*inj*_ *E*_*inj*_ *e* ^−*i*Δ*ωt*^, where Δ*ω* = *ω*_*inj*_ − *ω*, with *ω*_*inj*_ as the injected angular frequency and *ω* as the free-running angular frequency of the total electric field of the system in the absence of injection, *E*_*inj*_ as the injected field, and *k*_*inj*_ as a coupling rate for the injected signal. Such a laser system can be described by the following dimensionless rate equations (see Supplementary Equations for a derivation):

1 $$\frac{d{Y}_{A}}{dt}=\frac{1}{2{\tau }_{p}}({M}_{A}-1){Y}_{A}+{Y}_{B}({\eta }_{r}\,\sin \,\varphi -{\eta }_{i}\,\cos \,\varphi )+\frac{{K}_{inj}}{{\tau }_{N}}\,\cos \,{\varphi }_{A},$$

2 $$\frac{d{\varphi }_{A}}{dt}=\frac{{\alpha }_{H}}{2{\tau }_{p}}({M}_{A}-1)-(\omega -{{\rm{\Omega }}}_{A})-\frac{{Y}_{B}}{{Y}_{A}}({\eta }_{r}\,\cos \,\varphi +{\eta }_{i}\,\sin \,\varphi )-\frac{{K}_{inj}}{{\tau }_{N}{Y}_{A}}\,\sin \,{\varphi }_{A}-{\rm{\Delta }}\omega ,$$

3 $$\frac{d{Y}_{B}}{dt}=\frac{1}{2{\tau }_{p}}({M}_{B}-1){Y}_{B}-{Y}_{A}({\eta }_{r}\,\sin \,\varphi +{\eta }_{i}\,\cos \,\varphi ),$$

4 $$\frac{d{\varphi }_{B}}{dt}=\frac{{\alpha }_{H}}{2{\tau }_{p}}({M}_{B}-1)-(\omega -{{\rm{\Omega }}}_{B})-\frac{{Y}_{A}}{{Y}_{B}}({\eta }_{r}\,\cos \,\varphi -{\eta }_{i}\,\sin \,\varphi )-{\rm{\Delta }}\omega ,$$

5 $$\frac{d{M}_{A,B}}{dt}=\frac{1}{{\tau }_{N}}[{Q}_{A,B}-{M}_{A,B}(1+{Y}_{A,B}^{2})]\mathrm{.}$$

One can also note that either (2) or (4) can be replaced by an equivalent rate equation for the phase difference *ϕ* (=*ϕ*_*B*_ − *ϕ*_*A*_):

6 $$\frac{d\varphi }{dt}=\frac{{\alpha }_{H}}{2{\tau }_{p}}({M}_{B}-{M}_{A})+{\rm{\Delta }}{\rm{\Omega }}-{\eta }_{r}\,\cos \,\varphi (\frac{{Y}_{A}}{{Y}_{B}}-\frac{{Y}_{B}}{{Y}_{A}})+{\eta }_{i}\,\sin \,\varphi (\frac{{Y}_{A}}{{Y}_{B}}+\frac{{Y}_{B}}{{Y}_{A}})+\frac{{K}_{inj}}{{\tau }_{N}{Y}_{A}}\,\sin \,{\varphi }_{A},$$

where Ω = Ω_*B*_ − Ω_*A*_ is the frequency offset between the cavity resonances of the two coupled lasers in the absence of injection, *Y*_*A*_, *Y*_*B*_ are the normalized fields, *ϕ*_*A*_, *ϕ*_*B*_ are the corresponding phases, and *M*_*A*_, *M*_*B*_ are the normalized carrier densities in guides A, B, respectively, *τ*_*N*_ is the carrier lifetime, *τ*_*p*_ is the photon lifetime, and *α*_*H*_ is the linewidth enhancement factor that accounts for the phase-amplitude coupling in the electric field. *η*_*r*_, *η*_*i*_ are the real and imaginary parts of the complex coupling coefficient *η*, which is mathematically defined as^12^

7 $$|\eta |={C}_{\eta }\exp (-2{W}_{r}\frac{d}{a}),{\rm{\arg }}(\eta )={C}_{\theta }-2{W}_{i}\frac{d}{a},$$

where *C*_*η*_, *C*_*θ*_ can be found from numerical integration and *W*_*r*_, *W*_*i*_ are the real and imaginary parts of the transverse propagation constant in the regions outside the cores of waveguides A and B. *Q*_*A*_, *Q*_*B*_ are normalized pumping rates for lasers A and B, defined by Eq. (S11) in Supplementary Document. Restricting attention in what follows to the case of equal pumping *Q* ≡ *Q*_*A*_ = *Q*_*B*_, these can be expressed in terms of the ratio of pumping rate to its threshold value, *P*/*P*_*th*_, as^12^

8 $$Q={C}_{Q}(\frac{P}{{P}_{th}}-1)+\frac{P}{{P}_{th}},$$

where ${C}_{Q}=\frac{{a}_{diff}}{{g}_{th}}{N}_{o}$, with *a*_*diff*_ as the differential gain, *N*_*o*_ as the carrier density at transparency and *g*_*th*_ as the gain per unit length at threshold. *K*_*inj*_ represents the dimensionless injection level and is mathematically defined as

9 $${K}_{inj}=\sqrt{\frac{c{a}_{diff}{\tau }_{N}}{n}}{k}_{inj}{E}_{inj}{\tau }_{N},$$

where *n* is the refractive index. As in our recent work^12^, the following set of parameter values is considered: *a* = 4 *μ*m, *a*_*diff*_ = 1 × 10^−15^ cm^2^, *N*_*o*_ = 1 × 10^18^ cm^−3^, *τ*_*N*_ = 1.0 ns, *τ*_*p*_ = 1.53 ps, *n* = 3.4 and *P*/*P*_*th*_ = 2. We assume *α*_*H*_ = 2 unless otherwise specified. These values are typical for laterally-coupled semiconductor lasers. Table 1 gives numerical values of the key parameters for the four cases of interest that were analyzed in^12^. Here Δ*n*_*r*_ and Δ*n*_*i*_ are the real and imaginary parts of the index difference between the core and cladding regions of the waveguides that are used in calculating the transverse propagation constant and the coupling coefficient.

**Table 1:** Values of key parameters for modelling, using material parameter values given in text.

Δ*n*_*r*_	*g*_*th*_ (cm^−1^)	Δ*n*_*i*_	*W*_*r*_	*W*_*i*_	*C*_*Q*_	*C*_*η*_ (ns^−1^)	*C*_*θ*_ (rad)
0.00097	87.7	0	1.26	0	11.4	83.6	0
0.0005	90.6	0.000937	1.09	0.896	11.0	90.2	0.233
0	99.3	0.00103	0.795	1.22	10.1	91.9	0.294
−0.0005	108	0.00112	0.604	1.61	9.26	96.3	0.183

The cases shown in table were chosen in^12^ to illustrate a range of waveguide scenarios for comparative purposes; the first row considers the case of purely real index guiding, the second row refers to positive index guiding where some gain-guiding is also present, the third row is the case of no built-in index guiding (i.e. pure gain-guiding) and the last row simulates index antiguiding with gain-guiding.

### Numerical simulations

The dependence of the system dynamics on control parameters can be numerically investigated by integrating Eqs (1)–(6). To this end, we start our analysis by solving them using a fourth-order Runge-Kutta algorithm with a fixed time step of 1 ps. Each time series has been obtained by running the program over a time interval of 300 ns. To gain a complete view of the dynamics in the two-element laser array in the presence of external optical injection we construct the bifurcation map of dynamical regimes of the system in the parameter plane of the injection ratio *K* and optical frequency detuning Δ*f*(=(Δ*ω*_*inj*_)/(2*π*)); see the section of asymptotic analysis for the definition of *K* and Δ*ω*_*inj*_.

We first consider locking in the single laser case. If *η* → 0, Eqs (1)–(6) reduce to the well-known equations of a single laser with optical injection^39^. Figure 1 displays the typical stability map of different regions in the (*K*, Δ*f*)-plane, with each region corresponding to a different dynamic behavior. A 400 × 400 grid was used to discretize a square region of the parameter space. The color coding corresponds to the different dynamic regimes identified by a bifurcation analysis, i.e. from the extrema of the laser intensity time series, in which cw, period one (P1), period two (P2), and complex dynamics are identified as a constant intensity, two intensity extrema, four intensity extrema, and even more extrema, respectively. The regular dynamics including cw, P1, and P2 are marked in white, dark blue, and light blue, respectively. Qualitatively, we define complicated dynamics including chaos where the number of extrema exceeds four and use gradually changing colors from green to yellow, red, and dark red, to represent them. In the white region, the laser is injection-locked to the external light and thus operating in a cw state, which is the focus of the current study.

In fact, the (white) stable locking region is bounded by two types of bifurcations: one is an SN bifurcation and the other is referred to as a Hopf bifurcation, which is confirmed by using a path continuation technique (not shown here). As shown in Fig. 1, we present the locking region for four different representative values of the linewidth-enhancement factor *α*_*H*_. In the case of a zero value of *α*_*H*_, the stability map is symmetric about zero detuning and the stable locking region is bounded by SN and Hopf curves (one can get insight into them by using the path continuation technique^34^). In other non-zero *α*_*H*_ cases, however, the stability map appears asymmetric about zero detuning due to the amplitude-phase coupling in the field, and the stable locking region is shifted to large negative detunings for a large linewidth-enhancement factor (a larger scale of the vertical axis is used for *α*_*H*_ = 3 and 4). The locking region is determined by two SN boundaries for very small injection powers, and by an SN and a Hopf curve for moderate and strong injection levels. The evolution of the injection-locked solution and dynamics near these boundaries of the stable locking region has been extensively studied^34,39,40,50–53^. These reports can serve as a basis for studying injection locking phenomenon in laser arrays, which exhibit a very different and interesting behavior as will be discussed in the following.

**Figure 2:**
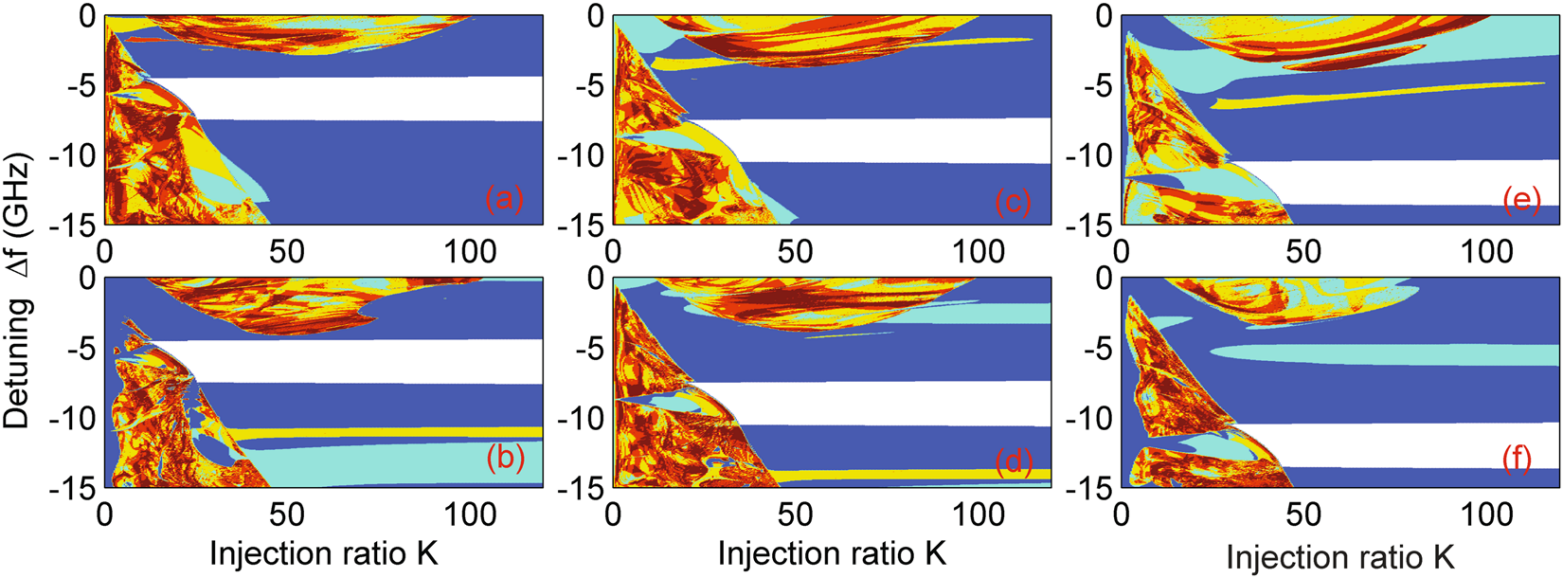
Stability map of the two-element array in the presence of injection in the (*K*, Δ*f*) plane for the case of the real index guide with Δ*n*_*r*_ = 0.000971, where *d*/*a* = 1.2 and *α*_*H*_ = 2. (**a**,**b**) offset ΔΩ/2*π* = −6, (**c,d**) −9, and (**e,f**) −12 GHz. The top row denotes Laser A, while the bottom row represents Laser B. The color codes are the same as those in Fig. 1.

Now we turn to study the locking behavior in the optically injected two-element array (*η* ≠ 0) and take the case of purely real index guiding with Δ*n*_*r*_ = 0.00971 (first row of Table 1) as an example. Figure 2 shows the stability map in this system for laser separation ratio *d*/*a* = 1.2, where three frequency offset values are considered: ΔΩ/2*π* = −6, −9, and −12 GHz. Again, the white area shows the well-defined region for which the injection-locked solutions can exist. Interestingly, the whole system (both lasers A and B in the two-element array) is locked to the external signal. It is worth noting that in one previous report single-mode spectra and narrow far-field lobs were obtained in a 10-element laser diode array injection-locked by a single-mode master laser^42^. However although the authors employed more elements in the laser array, their experimental results could be taken as evidence for locking all the elements at the same time.

Several other prominent phenomena can be identified as follows. First, a large variety of dynamics are seen in the (*K*, Δ*f*)-plane for the optically injected two-element array (however, we are only interested in the locking area in the current study). Second, the locking range differs notably from that in the case of a single laser subject to injection^39^: we find a nontrivial shape for the locking region and term it ‘trapezoid’. We find there are no qualitative changes in detuning along the axis of increasing injection ratio *K* (*x*-axis) after the system enters the locking range at a critical injection level (this can be approximately estimated from the SN boundary in the optically injected single laser case cf. Figs 1 and 2), and that the locking frequency range is symmetric about the frequency offset ΔΩ/2*π*. Third, the locking range can be tuned by controlling the offset ΔΩ/2*π*, while the trapezoid shape and width of the locking range are almost identical for different offset values, indicating that our results are extremely robust and, this control parameter has no obvious impact on the locking bandwidth in the two-element array case. In fact, to achieve locking one should be careful with the selection of the offset by choosing a value well below the Hopf bifurcation curve found in the case of a single laser subject to injection, otherwise the locking range will be interrupted by pulsations (see discussion of the final figure below).

When a positive offset ΔΩ/2*π* is chosen for finite *α*_*H*_, the optically injected two-element array cannot be locked to the external injection signal in the range of injection ratio considered. This is to be expected due to the asymmetric property introduced by the linewidth-enhancement factor in optically injected laser systems (see Supplementary Fig. S1 for more information).

### Asymptotic analysis of locking bandwidth

The conditions for the injection locking can be derived from the steady-state solutions of Eqs (1)–(6), which can be obtained by setting the left-hand sides (LHS) of these equations equal to zero. We consider the case of equal pumping in the lasers, i.e., *Q*_*A*_ = *Q*_*B*_ ≡ *Q*, and identical parameters for them except for taking into account the frequency offset. Based on the asymptotic analysis of the steady-state solutions and writing *η* = |*η*| exp (*i*Ψ), we find the locking condition satisfies the equation of the form (all details are given in Supplementary Document)

10 $${\rm{\Delta }}{\omega }_{inj}-{\rm{\Delta }}{\rm{\Omega }}=-\sqrt{1+{\alpha }_{H}^{2}}|\eta |\cos ({\rm{\Psi }}+{\varphi }_{s}+\theta )\frac{{Y}_{As}}{{Y}_{Bs}},$$

where the subscript ‘s’ denotes steady state, Δ*ω*_*inj*_ = *ω*_*inj*_ − Ω_*A*_ is the frequency detuning between the external injection field and the field of laser A, and tan*θ* = *α*_*H*_.

Neglecting terms of order *τ*_*p*_ *η*_*r*_, *τ*_*p*_ *η*_*i*_, and defining *K* = *K*_*inj*_/*Y*_*As*_ and Δ = 2*Kτ*_*p*_/*τ*_*N*_ < 1, we obtain an analytical expression for the ratio of the field amplitude in the two lasers

11 $$\frac{{Y}_{As}}{{Y}_{Bs}}\cong 1\pm \frac{Q{\rm{\Delta }}}{\pi (Q-1)},$$

Substituting (11) into (10) and considering the mean values of ±2/*π* for the cosine, we find an approximation for the locking condition given by

12 $$|{\rm{\Delta }}{\omega }_{inj}-{\rm{\Delta }}{\rm{\Omega }}|\le |\eta |\sqrt{1+{\alpha }_{H}^{2}}[1\pm K\frac{2}{\pi }\frac{{\tau }_{p}}{{\tau }_{N}}\frac{Q}{(Q-1)}]\mathrm{.}$$

**Figure 3:**
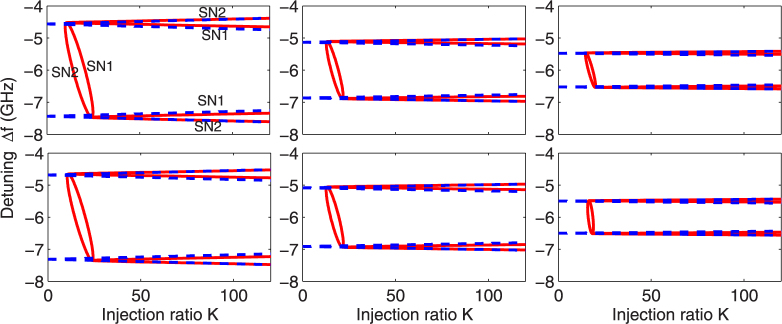
Bifurcation diagram of the two-element array in the presence of injection in the (*K*, Δ*f*)-plane, where ΔΩ/2*π* = −6 GHz and *α*_*H*_ = 2. (top row) real index guide with Δ*n*_*r*_ = 0.000971: (left to right) *d*/*a* = 1.2, *d*/*a* = 1.4, and *d*/*a* = 1.6; (bottom row) real index antiguide with Δ*n*_*r*_ = −0.0005 and gain-guiding: (left to right) *d*/*a* = 2.7, *d*/*a* = 3.0, and *d*/*a* = 3.5. Solid line: simulation; Broken line: approximation using Eq. (12).

It is worth noting that 1) this approximation corresponds to the branches of SN (also called fold or limit point) bifurcations existing in the laterally-coupled lasers subject to optical injection, which can be confirmed by carrying out linear stability analysis; 2) the locking range confined by these SN lines only weakly depends on the injection ratio *K*, which confirms the results in Figs 2 and 3) stable locking occurs in the whole range under the condition that lasers A and B are only weakly coupled. One example is shown in Fig. 2, where both the lower and upper limits are identified as SN bifurcation curves with the aid of our asymptotic analysis, and no unstable state is found between them. However, part of the locking range in the (*K*, Δ*f*)-plane may be dynamically unstable if the coupling strength between the lasers A and B is not weak enough. Since we consider only the weak coupling case in accordance with the validity of coupled-mode theory, stable cw operation can be guaranteed in the whole locking range in the (*K*, Δ*f*)-plane. For the sake of simplicity, for not too large *K*, the final term on the right hand side (RHS) of (12) is sufficiently small, then a good approximation is

13 $$|{\rm{\Delta }}{\omega }_{inj}-{\rm{\Delta }}{\rm{\Omega }}|\le |\eta |\sqrt{1+{\alpha }_{H}^{2}}\mathrm{.}$$

This result can be compared with the well-known corresponding result for a single laser subject to optical injection^39^:

14 $$|{\rm{\Delta }}{\omega }_{inj}|\le \frac{K}{{\tau }_{N}}\sqrt{1+{\alpha }_{H}^{2}}\mathrm{.}$$

It is worth noting that, as expected, the negative detuning part of Eq. (14) is clearly seen in the plots of Fig. 1, but also that an amended form of this result is seen to form the lower left stability boundary of the plots in Fig. 2. For more details of the latter, please see the Supplementary Document.

Returning to Eq. (13) for the coupled laser system, it follows that the approximate bandwidth (BW) of the locking region is given by

15 $$BW\cong \frac{|\eta |}{\pi }\sqrt{1+{\alpha }_{H}^{2}}\,(Hz)\mathrm{.}$$

Eqs (12), (13) and (15) are the main mathematical results of this paper. It is interesting to find that the locking bandwidth can be approximated by such a simple analytical expression, where the control parameters are coupling strength and the linewidth enhancement factor. Additionally, it should note that the locking range given by Eqs (12) or (13) is symmetric with respect to the condition Δ*ω*_*inj*_ = ΔΩ, which explains our earlier observation about the numerical results in Fig. 2.

In the next section, we illustrate and compare our approximate results with simulations.

### Validating the approximation

In order to explore the validity of our asymptotic approximations, we have determined numerically the SN-bifurcation points from the equations and followed them using AUTO continuation software^49^. In our previous work, we have shown that the waveguide structures are of crucial importance to determine the dynamics and phase-locked regions in the stand-alone two-element laser arrays^12^. We have to understand the influence of waveguide structures on the locking phenomenon so as to draw a generic conclusion. To this end, we have compared the results for four cases as mentioned above, i.e., (1) real index guide with Δ*n*_*r*_ = 0.000971, (2) real index guide with Δ*n*_*r*_ = 0.0005 and gain-guiding, (3) guide with Δ*n*_*r*_ = 0, pure gain-guiding, and (4) real index antiguide with Δ*n*_*r*_ = −0.0005 and gain-guiding. The corresponding parameters have been detailed in Table 1. Moreover, the influence of the laser separation ratio *d*/*a* has been taken into account in this section.

Figure 3 shows the boundaries of the stability region, i.e., the SN bifurcations, given in Eq. (12), depending on *d*/*a* and for two different waveguide parameters (i.e., real index guide with Δ*n*_*r*_ = 0.000971 and real index antiguide with Δ*n*_*r*_ = −0.0005 and gain-guiding; see Supplementary Fig. S2 for real index guide with Δ*n*_*r*_ = 0.0005 and gain-guiding, as well as guide with Δ*n*_*r*_ = 0, pure gain-guiding). The numerical results obtained by the path continuation method are used to validate the asymptotic results. The broken lines give the approximate results and the solid curves give the numerical continuation results. As can be seen from this figure and Supplementary Fig. S2, our asymptotic approximation is in good agreement with the numerical results in all cases, which verifies the accuracy of our approximate results. Other observed results are summarized as follows. First, the shape of the locking region remains almost the same in the four waveguide structures and as the laser separation is changed. Second, there always exist two SN braches (SN1 and SN2) that (1) become separated for small laser separation and large injection ratio *K*, (2) are almost superposed for other cases and (3) cross each other twice at two small values of *K*, forming an ellipse (red) which is located at the left hand side of the trapezoid structure. Third, the numerical results obtained by the path continuation method show that these two branches belong to the SN bifurcations occurring on two different equilibrium solutions, which were assumed to be symmetric and asymmetric solutions in^48^. Even though the difference between supercritical and subcritical parts of SN bifurcations is not indicated in this figure, we should note that the outer branch (SN2) which is almost parallel in the horizontal (*x*) axis is always supercritical. This corresponds to the existence of stable stationary states between them. However, outside the symmetric branch, no stationary solutions exist and the two-element laser array exhibits pulsations and other complicated behaviours via other further bifurcations. One can formulate the linearized problem from Eqs (1)–(6), derive the characteristic equation for the growth rate, and finally determine the stability of the steady state using Routh-Hurwitz conditions^12,52,54,55^. However, we have used continuation package AUTO to determine the supercritical or subcritical property, and one example will be shown at the end of this section.

It is interesting to note that in our system the stable locking is bounded only by SN curves for these weak coupling levels studied, which is distinctively different from (1) the single laser with injection whose locking region is bounded by an SN line and a Hopf bifurcation line^34,39^, and (2) three-element laser arrays in the absence of injection where SN, Hopf, and transcritical bifurcations delimit the locking boundaries^27,56,57^. This implies that the locking behavior presented in the two-element laser array in the presence of injection has a different origin than the one encountered in previous systems.

On the other hand, in Fig. 3, one can clearly observe that the width of the locking region bounded by the SN branches strongly depends on the laser separation ratio *d*/*a*. When this control parameter is increased progressively, the locking region shrinks in size. We will inspect the dependence of the locking bandwidth on *d*/*a* in more detail later in this section. Additionally, the red ellipse in each subfigure represents the threshold of stability for the injection ratio *K*: the injected two-element laser array is unstable on the left of the ellipse, whereas the whole system is locked to the injection on its right. This can be roughly approximated by Eq. (S45) (see Supplementary Document; the result for a single laser subject to injection), which, however, is not indicated in Fig. 3.

**Figure 4:**
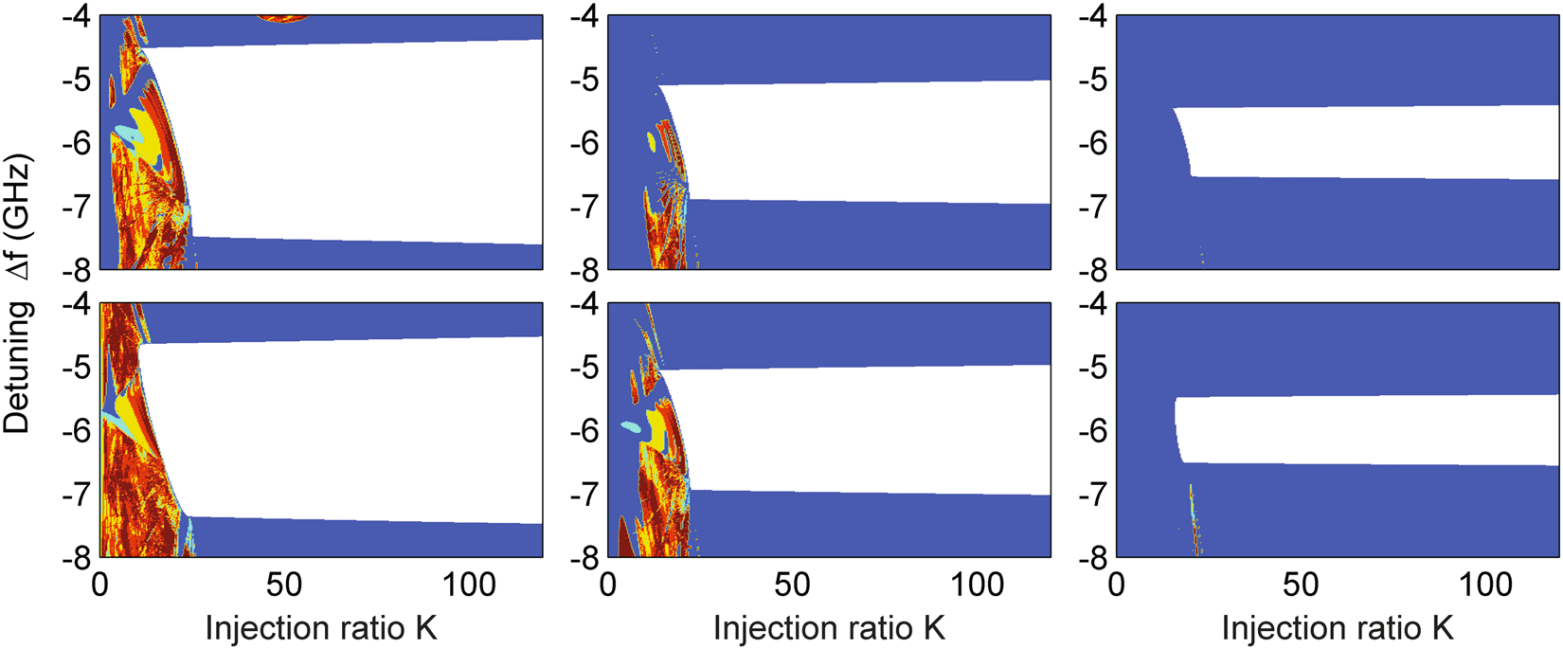
Bifurcation diagram of the two-element array in the presence of injection in the (*K*, Δ*f*)-plane, where ΔΩ/2*π* = −6 GHz and *α*_*H*_ = 2. Parameters are the same as those in Fig. 3. The results are presented only for Laser B. The color codes are the same as those in Fig. 1.

We further examine the stability properties by direct numerical integration of the dimensionless rate Eqs (1)–(6) and obtain the corresponding stability map of various dynamical regimes using the same color coding as Fig. 1. Since identical locking regions are found for lasers A and B, only the results for laser B are shown in Fig. 4. Again, only the results for real index guide with Δ*n*_*r*_ = 0.000971 and real index antiguide with Δ*n*_*r*_ = −0.0005 and gain-guiding are displayed here; see Supplementary Fig. S3 for real index guide with Δ*n*_*r*_ = 0.0005 and gain-guiding, as well as guide with Δ*n*_*r*_ = 0, pure gain-guiding. By comparison with Fig. 3 we notice that the stability region bounded by the SN bifurcation curves only admits the injection-locked solutions in the two-element laser array with injection. Moreover, the trend of the locking width versus *d*/*a* is confirmed by the time-dependent results.

**Figure 5:**
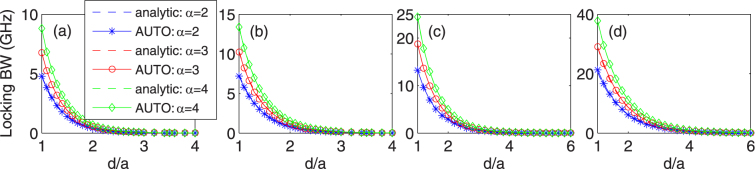
Locking bandwidth as a function of *d*/*a* four different representative values of the linewidth-enhancement factor *α*_*H*_. (**a**) Δ*n*_*r*_ = 0.000971, (**b**) 0.0005, (**c**) 0.0, and (**d**) −0.0005; Solid line: simulation; Broken line: approximation using Eq. (15).

Next we consider the variation of the locking bandwidth with the laser separation ratio *d*/*a* and the linewidth enhancement factor *α*_*H*_. Here the bandwidth is defined as the interval of the frequency detuning, where the stable locked solutions exist, i.e., the distance between the lower and upper limits for the frequency detuning bounded by two supercritical parts of SN bifurcation branches (in the vertical direction on the maps). An approximation of the bandwidth is given by Eq. (15). Figure 5 represents the exact and approximate bandwidth calculated at *K* = 60 in the four cases of waveguide parameters. The solid lines show the numerical simulations, while broken curves represent the analytic results. As can be seen, the analytical bandwidth is always in excellent agreement with the numerical simulations. Similar trend in the four waveguiding structures indicates that they have no restriction to the locking phenomenon in the two-element laser array in the presence of injection. Furthermore, it is noteworthy that the bandwidth weakly depends on the value of the injection ratio *K*, and in Eq. (15) such dependence is neglected. When the bandwidth is calculated at a larger value of *K*, say 100, the exact bandwidth should be slightly larger than the approximate result given by Eq. (15), especially for small values of *d*/*a* (cf. Fig. 3). High accuracy, however, can be maintained regardless of the values of *d*/*a* if the analytic expression, given by Eq. (12), is used for the determination of the approximate bandwidth.

In Fig. 5, when the laser separation ratio *d*/*a* is increased, the locking bandwidth decreases roughly exponentially and finally drops to zero. There is a general trend of larger locking bandwidth for larger values of the linewidth enhancement factor. This is expected because from Eq. (15), one can see that the locking bandwidth is proportional to |*η*| and $\sqrt{1+{\alpha }_{H}^{2}}$. Larger *d*/*a* means smaller |*η*|, resulting in smaller bandwidth. When the laser separation is too large, the interaction between the two laterally-coupled lasers is negligible and the injected light is insufficient to lock the whole system at the same time. This indicates that the corresponding SN bifurcation boundaries of stationary solutions disappear. In this case, the system reduces to a situation very similar to that in a single laser subject to optical injection^34,39^; see Fig. 1 for the corresponding locking range.

**Figure 6:**
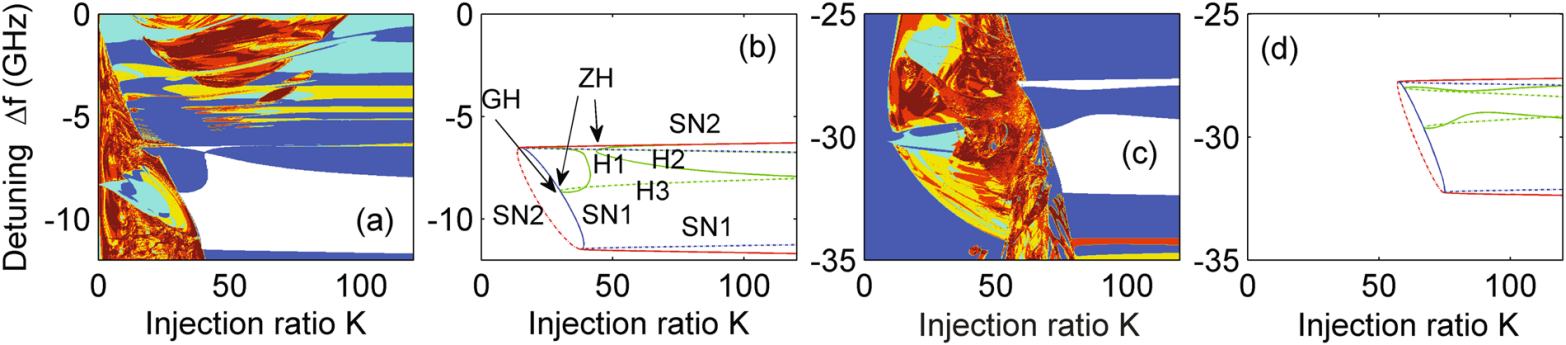
Stability map and bifurcation diagram of the two-element array in the presence of injection in the (*K*, Δ*f*)-plane for the case of the real index guide with Δ*n*_*r*_ = 0.000971. The stability map is obtained from the intensity time traces of Laser B. (**a,b**) *α*_*H*_ = 2, *d*/*a* = 1.0, and ΔΩ/2*π* = −9 GHz; (**c,d**) *α*_*H*_ = 3, *d*/*a* = 1.2, and ΔΩ/2*π* = −30 GHz. In (**b**) and (**d**), blue stands for SN1, red for SN2, and green for Hopf bifurcations; solid line denotes supercritical, while broken line represents subcritical. In (**b**), the branches of Hopf bifurcations H1 and H2 are supercritical, while H3 is subcritical. Codimension-2 bifurcation points including generalized Hopf (GH) and zero-Hopf (ZH) are indicated; in (**d**), the codimension-1 bifurcation curves and codimension-2 bifurcation points have the same definitions as those in (**b**), so they are not indicated for simplicity.

Finally, we emphasize that part of the locking range may be dynamically unstable for small laser separation ratio *d*/*a* and hence higher evanescent coupling. A typical example is shown in Fig. 6(a), where a real index guide with Δ*n*_*r*_ = 0.000971, offset ΔΩ/2*π* = −9 GHz and *α*_*H*_ = 2 is considered. One can see that part of the injection locking range is indeed interrupted by self-pulsation oscillations due to supercritical Hopf bifurcations (H1 and H2) [see Fig. 6(a)]. In the bifurcation diagram of Fig. 6(b), the solid and broken curves represent the supercritical and subcritical cases, respectively. The change in stability of the Hopf bifurcations is found at codimension-2 bifurcation points, i.e., generalized Hopf (GH) bifurcation on H1and H3 branches and zero-Hopf (ZH) bifurcation on H2 branch. In particular, at a GH point, the supercritical Hopf bifurcation becomes subcritical; at a ZH point, SN and Hopf curves are tangent and they change from supercritical to subcritical^34^. These are clearly indicated in this diagram. To further test this, we present another example for larger offset ΔΩ/2*π* = −30 GHz and *α*_*H*_ = 3 in Fig. 6(c). It is worth noting the trend of needing high injection ratio to achieve locking with increasing frequency offset. However a qualitative comparison with the bifurcation diagram in Fig. 6(d) shows a similar more complex division of the stable locking region. This is a general phenomenon concerning the behavior with high evanescent coupling, regardless of the values of the offset and *α*_*H*_. This is in contrast with weak evanescent coupling where a change of stability only occurs through an SN bifurcation, given by Eq. (12). Moreover, the mathematical investigation of all Hopf branches in the two-element laser array in the presence of injection is much more complicated compared to that in a single laser with injection, but deserves a detailed comparison with simulations (see, for example^54^) and this will be addressed in future work.

## Discussion

In the present work we have studied a model for an optically injected two-element laser array where two lasers are laterally coupled and one of them is subject to optical injection. In particular, the locking range and bandwidth have been investigated analytically and numerically. Approximate analytical results, using the derived asymptotic expression, of the parameter range where injection locking is expected to occur in the plane of the injection ratio against the frequency detuning agree very well with the numerical results. Our analysis has shown that the behavior of the injection-locked array solution is different from that for injection-locking of single lasers^39^ and from that found in three laterally-coupled lasers in the absence of injection^27^. The locked state occurs in a stable locking region bounded by two supercritical, almost parallel, SN lines. The locking bandwidth largely depends on the linewidth enhancement factor *α*_*H*_ and the laser separation ratio *d*/*a*: larger *α*_*H*_ and smaller *d*/*a* lead to larger locking bandwidth. Moreover, the locking range can be tuned readily by controlling the frequency offset between the two laterally-coupled lasers. Our results are presented for four different waveguiding structures including purely real index guiding, pure gain-guiding, and combinations of index guiding and antiguiding with gain-guiding which differ in their behaviour in terms of the variation of coupling amplitude and phase with device separation^12^. Even though it has been demonstrated that these structures have a significant influence on the dynamics of two laterally-coupled lasers^12^, similar injection-locking behaviours are seen when optical injection is applied to one laser of this model. Since we show for a pair of weakly coupled devices that injection into just one can influence the behaviour of the other, it is interesting to postulate how this might be extended to arrays with multiply coupled devices. Examples of this already exist where the tendency has been on use of injection for controlling the spatial field profiles of the whole VCSEL array^46,47^ where devices are strongly coupled. In contrast our concentration is on weak coupling where any influence may extend to only part of an array; hence the nature of this provides an aspect of interest for future work.

## Methods

In this contribution, we have employed combined methods to study the locking conditions and bandwidth of two laterally-coupled semiconductor lasers in the presence of external optical injection. Here we briefly summarize these methods.

### Direct numerical simulations

The rate Eqs (1)–(6) have been integrated by using a fourth-order Runge-Kutta algorithm. Specifically, each time series has been obtained by running the program with a fixed time step of 1 ps over a time interval of 300 ns. We have carried out a comprehensive bifurcation analysis and constructed high-resolution two-dimensional maps to show phased-locked regions and other dynamical regions.

### Numerical path continuation

The dynamics of the proposed system has been explored using AUTO software (standard numerical path continuation package). This allows tracking of the stable or unstable steady-state and periodic solutions and detection of various bifurcations. In particular, we focus on the principal saddle-node and Hopf bifurcations. A saddle-node bifurcation is associated with the appearance of one zero eigenvalue, which indicates a collision and disappearance of two equilibria in dynamical systems; a Hopf bifurcation corresponds to the presence of a pair of purely imaginary eigenvalues, which indicates the birth of a limit cycle from an equilibrium in dynamical systems. Both of them can be subcritical or supercritical and reveal insights into the boundaries of locking regions^58^.

### Asymptotic analysis

We have derived the conditions for injection locking from the steady-state solutions of Eqs (1)–(6). We have obtained a simple mathematical expression that accounts for a nontrivial trapezoidal region, where the two-element laser array subject to optical injection admits stably injection-locked states. The details are presented in the Supplementary Document. The validity of these asymptotic approximations has been confirmed using direct numerical simulations and numerical path continuation methods.

## Electronic supplementary material

Supplementary Document
