# Supplementary material for: Locking bandwidth of two laterally-coupled semiconductor lasers subject to optical injection
Source: Sci Rep. 2018 Jan 8;8:109. doi: 10.1038/s41598-017-18379-7 (PMC5758791; doi:10.1038/s41598-017-18379-7)
Supplement: Supplementary file 1 — Supplementary Document [file 41598_2017_18379_MOESM1_ESM.pdf]

Locking bandwidth of two laterally-coupled semiconductor lasers subject to optical injection

Nianqiang Li[1], H. Susanto[2], B. R. Cemlyn[1], I. D. Henning[1], and M. J. Adams[1,*]

[1]School of Computer Science and Electronic Engineering, University of Essex, Wivenhoe Park, Colchester CO4 3SQ, United Kingdom
[2]Department of Mathematical Sciences, University of Essex, Wivenhoe Park, Colchester CO4 3SQ, United Kingdom
[*]adamm@essex.ac.uk

ABSTRACT

This document provides supplementary information to “Locking bandwidth of two laterally-coupled semiconductor lasers subject to optical injection,”. Here we summarize the mathematical details of the derivation of the rate equations and of steady-solutions and locking conditions, which are of vital importance for readers to understand our mathematical results. Some additional figures are presented as well.

Supplementary Information

Derivation of the rate equations for coupled lasers with optical injection

Equations describing the time evolution of electric fields in coupled lasers A and B in the absence of optical injection are given by (A19) and (A20) of[1]:

$$\frac{dE_A}{dt} = \Gamma\frac{c}{2n_g}a_{diff}\left(N_A - N_{Ath}\right)\left(1 - i\alpha_H\right)E_A + i\left(\omega - \Omega_A\right)E_A + i\eta E_B. \tag{S1}$$

The corresponding rate equation for the field in waveguide B is given by

$$\frac{dE_B}{dt} = \Gamma\frac{c}{2n_g}a_{diff}\left(N_B - N_{Bth}\right)\left(1 - i\alpha_H\right)E_B + i\left(\omega - \Omega_B\right)E_B + i\eta E_A. \tag{S2}$$

In these equations E_A, E_B are the electric fields, N_A, N_B are the carrier concentrations with threshold values N_{Ath}, N_{Bth}, Γ is the optical confinement factor, a_{diff} is the differential gain, α_H is the linewidth enhancement factor, n_g is the group index, ω is the angular frequency of the total electric field, Ω_A, Ω_B are the individual cavity resonance angular frequencies, η is the coupling coefficient (expressed as a rate per unit time) and c is the speed of light.

Now allow a complex coupling coefficient $\eta = \eta_r + i\eta_i$ and add optical injection into the equation for laser A:

$$\frac{dE_A}{dt} = \Gamma\frac{c}{2n_g}a_{diff}\left(N_A - N_{Ath}\right)\left(1 - i\alpha_H\right)E_A + i\left(\omega - \Omega_A\right)E_A + i\left(\eta_r + i\eta_i\right)E_B + k_{inj}E_{inj}e^{-i\Delta\omega t}, \tag{S3}$$

where $\Delta\omega = \omega_{inj} - \omega$, with ω_{inj} as the injected frequency, E_{inj} as the injected field, and k_{inj} as a coupling rate for the injected signal.

Writing $E_{A,B} = X_{A,B}\exp\left(-i\phi_{A,B} - i\Delta\omega t\right)$ and $\phi = \phi_B - \phi_A$, equations (S3) and (S2) become

$$\frac{dX_A}{dt} = \Gamma\frac{c}{2n_g}a_{diff}\left(N_A - N_{Ath}\right)X_A + X_B\left(\eta_r\sin\phi - \eta_i\cos\phi\right) + k_{inj}E_{inj}\cos\phi_A, \quad \text{(S4)}$$

$$\frac{d\phi_A}{dt} = \alpha_H\Gamma\frac{c}{2n_g}a_{diff}\left(N_A - N_{Ath}\right) - \left(\omega - \Omega_A\right) - \frac{X_B}{X_A}\left(\eta_r\cos\phi + \eta_i\sin\phi\right) - \frac{k_{inj}E_{inj}}{X_A}\sin\phi_A - \Delta\omega, \quad \text{(S5)}$$

$$\frac{dX_B}{dt} = \Gamma\frac{c}{2n_g}a_{diff}\left(N_B - N_{Bth}\right)X_B - X_A\left(\eta_r\sin\phi + \eta_i\cos\phi\right), \quad \text{(S6)}$$

$$\frac{d\phi_B}{dt} = \alpha_H\Gamma\frac{c}{2n_g}a_{diff}\left(N_B - N_{Bth}\right) - \left(\omega - \Omega_B\right) - \frac{X_A}{X_B}\left(\eta_r\cos\phi - \eta_i\sin\phi\right) - \Delta\omega. \quad \text{(S7)}$$

The rate equations for carrier concentrations N_A, N_B are given by (A24) of[1]:

$$\frac{dN_{A,B}}{dt} = P_{A,B} - \frac{N_{A,B}}{\tau_N} - \frac{c}{n}\left[g_{A,Bth} + a_{diff}\left(N_{A,B} - N_{A,Bth}\right)\right]X_{A,B}^2, \quad \text{(S8)}$$

where $P_{A,B}$ is the pumping rate (dimensions $L^{-3}T^{-1}$), τ_N is the carrier lifetime, n is the refractive index and $g_{A,Bth}$ is the threshold gain per unit length of laser A, B. Since the two lasers are identical (except for their resonant frequencies), it follows that the values of threshold gain are the same and given by

$$g_{Ath} \equiv g_{Bth} = \frac{n_g}{\Gamma c\tau_p} \quad \text{(S9)}$$

where τ_p is the photon lifetime.

Note that so far the variables are not normalized, so that the dimensions of N_A, N_B are L^{-3}, the fields have dimensions $L^{-3/2}$, and the gain and loss have dimension L^{-1}. Now define the dimensionless variables:

$$M_{A,B} = 1 + \frac{c}{n_g}\Gamma a_{diff}\tau_p\left(N_{A,B} - N_{A,Bth}\right), \quad \text{(S10)}$$

$$Q_{A,B} = 1 + \frac{c}{n_g}\Gamma a_{diff}\tau_p\left(P_{A,B}\tau_N - N_{A,Bth}\right), \quad \text{(S11)}$$

$$Y_{A,B} = \sqrt{\frac{ca_{diff}\tau_N}{n}}X_{A,B}, \quad \text{(S12)}$$

$$K_{inj} = \sqrt{\frac{ca_{diff}\tau_N}{n}}k_{inj}E_{inj}\tau_N. \quad \text{(S13)}$$

In terms of these variables, equations (S4)-(S8) become

$$\frac{dY_A}{dt} = \frac{1}{2\tau_p}\left(M_A - 1\right)Y_A + Y_B\left(\eta_r\sin\phi - \eta_i\cos\phi\right) + \frac{K_{inj}}{\tau_N}\cos\phi_A, \quad \text{(S14)}$$

$$\frac{d\phi_A}{dt} = \frac{\alpha_H}{2\tau_p}\left(M_A - 1\right) - \left(\omega - \Omega_A\right) - \frac{Y_B}{Y_A}\left(\eta_r\cos\phi + \eta_i\sin\phi\right) - \frac{K_{inj}}{\tau_N Y_A}\sin\phi_A - \Delta\omega, \quad \text{(S15)}$$

$$\frac{dY_B}{dt} = \frac{1}{2\tau_p}\left(M_B - 1\right)Y_B - Y_A\left(\eta_r\sin\phi + \eta_i\cos\phi\right), \quad \text{(S16)}$$

$$\frac{d\phi_B}{dt} = \frac{\alpha_H}{2\tau_p}\left(M_B - 1\right) - \left(\omega - \Omega_B\right) - \frac{Y_A}{Y_B}\left(\eta_r\cos\phi - \eta_i\sin\phi\right) - \Delta\omega, \quad \text{(S17)}$$

$$\frac{dM_{A,B}}{dt} = \frac{1}{\tau_N}\left[Q_{A,B} - M_{A,B}\left(1 + Y_{A,B}^2\right)\right]. \quad \text{(S18)}$$

It is also possible to replace either (S15) or (S17) with an equivalent rate equation for the phase difference ϕ:

$$\frac{d\phi}{dt} = \frac{\alpha_H}{2\tau_p}\left(M_B - M_A\right) + \Delta\Omega - \eta_r\cos\phi\left(\frac{Y_A}{Y_B} - \frac{Y_B}{Y_A}\right) + \eta_i\sin\phi\left(\frac{Y_A}{Y_B} + \frac{Y_B}{Y_A}\right) + \frac{K_{inj}}{\tau_N Y_A}\sin\phi_A, \quad \text{(S19)}$$

where $\Omega = \Omega_B - \Omega_A$ is the detuning between the cavity resonances of the two lasers.

Steady-sate solutions and locking conditions

The steady-state solutions of (S14)-(S18) for the case of equal pumping in the lasers ($Q_A = Q_B \equiv Q$) are

$$M_{As} = -1 - 2\tau_p\left(\eta_r \sin\phi_s - \eta_i \cos\phi_s\right)\frac{Y_{Bs}}{Y_A s} - 2\frac{\tau_p}{\tau_N}K\cos\phi_{As}, \tag{S20}$$

$$M_{Bs} = 1 + 2\tau_p\left(\eta_r \sin\phi_s + \eta_i \cos\phi_s\right)\frac{Y_{As}}{Y_B s}, \tag{S21}$$

$$Y_{As}^2 = \frac{Q}{1 - 2\tau_p\left(\eta_r \sin\phi_s - \eta_i \cos\phi_s\right)\frac{Y_{Bs}}{Y_A s} - 2\frac{\tau_p}{\tau_N}K\cos\phi_{As}} - 1, \tag{S22}$$

$$Y_{Bs}^2 = \frac{Q}{1 + 2\tau_p\left(\eta_r \sin\phi_s + \eta_i \cos\phi_s\right)\frac{Y_{As}}{Y_B s}} - 1, \tag{S23}$$

$$\alpha_H\left(M_{As} - 1\right) = 2\tau_p\left(\omega - \Omega_A\right) + 2\tau_p\left(\eta_r \cos\phi_s + \eta_i \sin\phi_s\right)\frac{Y_{Bs}}{Y_{As}} + 2\frac{\tau_p}{\tau_N}K\sin\phi_{As} + 2\tau_p\Delta\omega, \tag{S24}$$

$$\alpha_H\left(M_{Bs} - 1\right) = 2\tau_p\left(\omega - \Omega_B\right) + 2\tau_p\left(\eta_r \cos\phi_s - \eta_i \sin\phi_s\right)\frac{Y_{As}}{Y_{Bs}} + 2\tau_p\Delta\omega, \tag{S25}$$

where $K = K_{inj}/Y_{As}$. Neglecting terms of order $\tau_p\eta_r$, $\tau_p\eta_i$ in (S22) and (S23), and defining $\Delta = 2K\tau_p/\tau_N < 1$, yields

$$Y_{As}^2 \cong \frac{Q}{1 - \Delta\cos\phi_{As}} - 1, \tag{S26}$$

$$Y_{Bs}^2 \cong Q - 1, \tag{S27}$$

$$\left(\frac{Y_{As}}{Y_{Bs}}\right)^2 \cong \frac{Q - 1 + \Delta\cos\phi_{As}}{\left(Q-1\right)\left(1 - \Delta\cos\phi_{As}\right)} = 1 + \frac{Q\Delta\cos\phi_{As}}{\left(Q-1\right)\left(1 - \Delta\cos\phi_{As}\right)}. \tag{S28}$$

Substituting for M_{Bs} from (S21) in (S25) yields

$$\alpha_H\left(\eta_r \sin\phi_s + \eta_i \cos\phi_s\right)\frac{Y_{As}}{Y_{Bs}} = \left(\omega - \Omega_B\right) + \left(\eta_r \cos\phi_s - \eta_i \sin\phi_s\right)\frac{Y_{As}}{Y_{Bs}} + \Delta\omega. \tag{S29}$$

Defining $\Delta\omega_{inj} = \omega_{inj} - \Omega_A$ and $\tan\theta = \alpha_H$, this equation can be rewritten in the form

$$\Delta\omega_{inj} - \Delta\Omega = \frac{Y_{As}}{Y_{Bs}}\left[\alpha_H\left(\eta_r \sin\phi_s + \eta_i \cos\phi_s\right) - \left(\eta_r \cos\phi_s - \eta_i \sin\phi_s\right)\right]. \tag{S30}$$

This can be more conveniently expressed as

$$\Delta\omega_{inj} - \Delta\Omega \cong \sqrt{1 + \alpha_H^2}\left[\eta_i \sin\left(\phi_s + \theta\right) - \eta_r \cos\left(\phi_s + \theta\right)\right]\frac{Y_{As}}{Y_{Bs}}. \tag{S31}$$

Substituting for M_{As} from (S20) in (S24) yields

$$-\alpha_H\left[\left(\eta_r \sin\phi_s - \eta_i \cos\phi_s\right)\frac{Y_{Bs}}{Y_{As}} + \frac{\Delta}{2\tau_p}\cos\phi_{As}\right] = \left(\omega - \Omega_A\right) + \left(\eta_r \cos\phi_s + \eta_i \sin\phi_s\right)\frac{Y_{Bs}}{Y_{As}} + \frac{\Delta}{2\tau_p}\sin\phi_{As} + \Delta\omega. \tag{S32}$$

This can be more conveniently expressed as

$$\Delta\omega_{inj} = -\sqrt{1 + \alpha_H^2}\left[\left(\eta_r \cos\left(\phi_s - \theta\right) + \eta_i \sin\left(\phi_s - \theta\right)\right)\frac{Y_{Bs}}{Y_{As}} + \frac{\Delta}{2\tau_p}\sin\left(\theta + \phi_{As}\right)\right]. \tag{S33}$$

Writing $\eta = |\eta|\exp\left(i\Psi\right)$, equations (S31) and (S33) may be written in the alternative forms

$$\Delta\omega_{inj} - \Delta\Omega = -\sqrt{1+\alpha_H^2}\,|\eta|\cos(\Psi+\phi_s+\theta)\,\frac{Y_{As}}{Y_{Bs}}, \tag{S34}$$

$$\Delta\omega_{inj} = -\sqrt{1+\alpha_H^2}\left[|\eta|\cos(\Psi-\phi_s+\theta)\,\frac{Y_{Bs}}{Y_{As}} + \frac{\Delta}{2\tau_p}\sin(\theta+\phi_{As})\right]. \tag{S35}$$

Equations (S34) and (S35) can be developed further by finding approximate expressions for the ratio of normalized fields Y_{AS}/Y_{BS}. To do this we expand the LHS of (S28) to first order in Δ:

$$\left(\frac{Y_{As}}{Y_{Bs}}\right)^2 \cong 1 + \frac{Q\Delta\cos\phi_{As}}{(Q-1)}. \tag{S36}$$

If we make the further assumption that $0 \le \phi_{As} \le \pi/2$, then the cosine in (S36) can be approximated by its mean value of $2/\pi$, so that

$$\frac{Y_{As}}{Y_{Bs}} \cong 1 + \frac{Q\Delta}{\pi(Q-1)}. \tag{S37}$$

Hence, substituting for Δ, a more useful version of (S34) is

$$\Delta\omega_{inj} - \Delta\Omega \cong -\sqrt{1+\alpha_H^2}\,|\eta|\cos(\Psi+\phi_s+\theta)\left[1+K\frac{2}{\pi}\frac{\tau_p}{\tau_N}\frac{Q}{(Q-1)}\right]. \tag{S38}$$

Making a similar procedure for (S35) yields

$$\Delta\omega_{inj} = -\sqrt{1+\alpha_H^2}\{|\eta|\cos(\Psi-\phi_s+\theta)\left[1-K\frac{2}{\pi}\frac{\tau_p}{\tau_N}\frac{Q}{(Q-1)}\right] + \frac{K}{\tau_N}\sin(\theta+\phi_{As})\}. \tag{S39}$$

It follows from (S38) and (S39) that the following conditions for locking apply

$$\left|\Delta\omega_{inj} - \Delta\Omega\right| \le |\eta|\sqrt{1+\alpha_H^2}\left[1+K\frac{2}{\pi}\frac{\tau_p}{\tau_N}\frac{Q}{(Q-1)}\right], \tag{S40}$$

$$\left|\Delta\omega_{inj}\right| \le \sqrt{1+\alpha_H^2}\{|\eta|\left[1-K\frac{2}{\pi}\frac{\tau_p}{\tau_N}\frac{Q}{(Q-1)}\right] + \frac{K}{\tau_N}\}. \tag{S41}$$

Returning to equation (S36), and now considering the case $\pi/2 \le \phi_{As} \le \pi$, the cosine in (S36) can be approximated by its mean value of $-2/\pi$. The corresponding locking conditions are

$$\left|\Delta\omega_{inj} - \Delta\Omega\right| \le |\eta|\sqrt{1+\alpha_H^2}\left[1-K\frac{2}{\pi}\frac{\tau_p}{\tau_N}\frac{Q}{(Q-1)}\right], \tag{S42}$$

$$\left|\Delta\omega_{inj}\right| \le \sqrt{1+\alpha_H^2}\{|\eta|\left[1+K\frac{2}{\pi}\frac{\tau_p}{\tau_N}\frac{Q}{(Q-1)}\right] + \frac{K}{\tau_N}\}. \tag{S43}$$

If the final term on the RHS of (S40,S42) is sufficiently small, then a good approximation is

$$\left|\omega_{inj} - \Omega_B\right| \le |\eta|\sqrt{1+\alpha_H^2}. \tag{S44}$$

In the limit $\eta_r \to 0$, equations (S41,S43) reduce to the well-known condition for a single laser subject to injection[2]:

$$\left|\Delta\omega_{inj}\right| \le \frac{K_{inj}}{\tau_N Y_{As}}\sqrt{1+\alpha_H^2}. \tag{S45}$$

Supplementary Figure S1

In Fig. S1 are examples of the stability maps for the offset of $\Delta\Omega/2\pi = 6$ GHz, and two different laser separation ratios; $d/a =$ 1.2 and 1.6. Here there are no stable locking regions, and both lasers operate in oscillatory states including periodic, quasi-periodic, and chaotic oscillations, in the whole $(K, \Delta f)$-plane considered. These are included for illustrative purposes to show that a wide range of behaviors are possible in this simple two laser system depending upon the choice of separation (coupling). Nevertheless these are not the focus of this study and thus we concentrate on the stable locking range and bandwidth in this study. In particular, we determine analytically the boundaries of stationary solutions and derive an approximate analytical expression for the locking range.

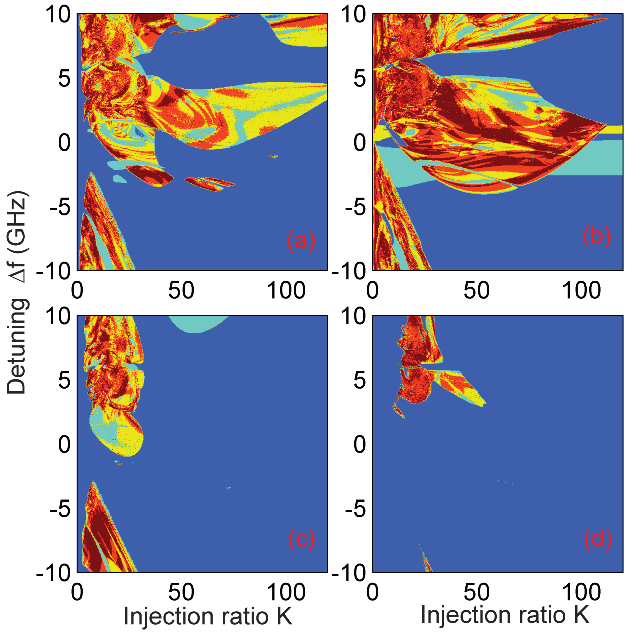

Figure S1. Stability map of the two-element array in the presence of injection in the $(K, \Delta f)$- plane for the case of the real index guide with $n_r = 0.000971$, where $\Delta\Omega/2\pi = 6$ GHz and $\alpha_H = 2$. (a,b) $d/a = 1.2$ and (c, d) 1.6. The left column denotes Laser A, while the right column represents Laser B. The color codes are the same as those in Fig. 1.

Supplementary Figure S2

Figure S2 shows the boundaries of the stability region, i.e., the SN bifurcations, given in Eq. (12), depending on d/a and for two different waveguide parameters, i.e., real index guide with $\Delta n_r = 0.0005$ and gain-guiding and Guide with $\Delta n_r = 0.0$, pure gain-guiding. Note that these are two other waveguide structures different from those included in the manuscript. This figure contains the results for asymptotic analysis and numerical path continuation methods. Figure S3 presents the corresponding simulation results obtained via direct integration of the rate equations.

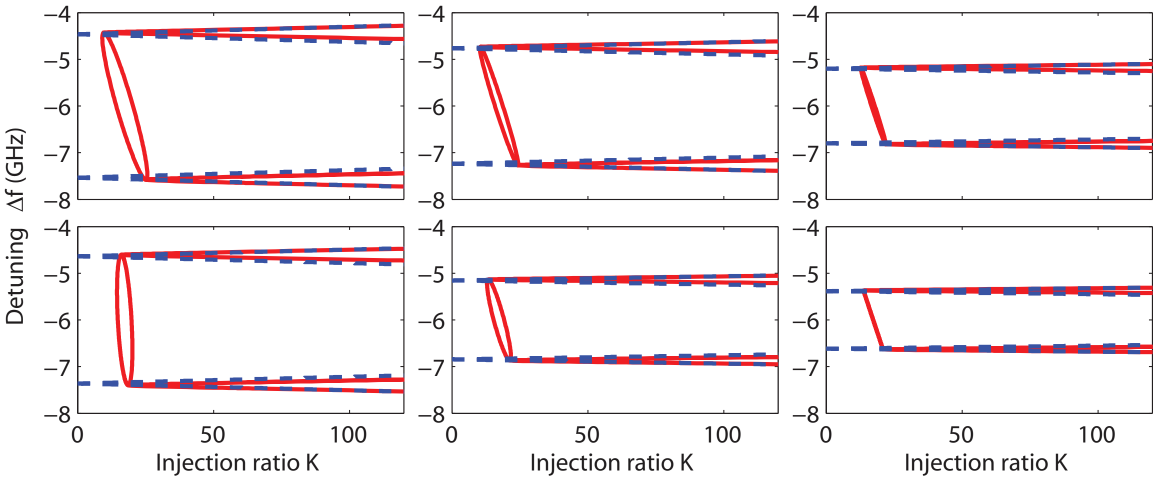

Figure S2. Bifurcation diagram of the two-element array in the presence of injection in the $(K, \Delta f)$-plane, where $\Delta\Omega/2\pi = -6$ GHz and $\alpha_H = 2$. (Top row) real index guide with $\Delta n_r = 0.0005$ and gain-guiding: (left to right) $d/a = 1.4$, $d/a = 1.5$, and $d/a = 1.7$; (bottom row) Guide with $\Delta n_r = 0.0$, pure gain-guiding: (left to right) $d/a = 2.0$, $d/a = 2.3$, and $d/a = 2.5$. Solid line: simulation; Broken line: approximation using Eq. (12).

Supplementary Figure S3

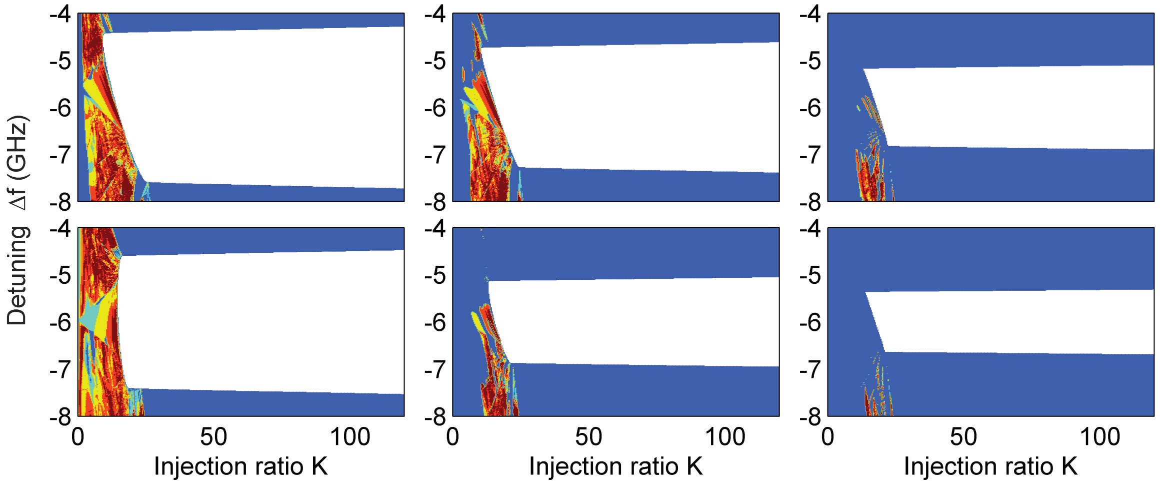

Figure S3. Bifurcation diagram of the two-element array in the presence of injection in the $(K, \Delta f)$-plane, where $\Delta\Omega/2\pi = -6$ GHz and $\alpha_H = 2$. Parameters are the same as those in Fig. S2. The results are presented only for Laser B. The color codes are the same as those in Fig. 1.

References

1. M. J. Adams, N. Li, B. R. Cemlyn, H. Susanto and I. D. Henning, "Effects of detuning, gain-guiding and index antiguiding on the dynamics of two laterally-coupled semiconductor lasers," Phys. Rev. A **95**, 053869 (2017).
2. F. Mogensen, H. Olesen and G. Jacobsen, "Locking conditions and stability properties for a semiconductor laser with external light injection," IEEE J. Quantum Electron. **QE-21**, 784-793 (1985).
